# Prolonged screen time is associated with increased severity of tic symptoms in children with tic disorders

**DOI:** 10.1186/s13052-025-01851-w

**Published:** 2025-01-26

**Authors:** Zakaria Ahmed Mohamed, Hanyu Dong, Yang Xue, Miaoshui Bai, Yuling Ouyang, Feiyong Jia

**Affiliations:** 1 Department of developmental and behavior pediatrics, Children’s Medical Center, The First Hospital of Jilin University, Jilin University, Changchun, China; 2 The Child Health Clinical Research Center of Jilin Province, Changchun, China

**Keywords:** Screen time, Tic disorders, YGTSS, Children

## Abstract

**Background:**

Recent studies have emphasized the association between prolonged screen exposure and neurodevelopmental disorders, though its correlation with tic disorders (TDs) remains ambiguous. We thus conducted this study to investigate the association between screen time (ST) and the severity of tic symptoms in children diagnosed with TDs.

**Methods:**

We conducted a retrospective case-control study with 342 cases of TDs and 270 controls, collecting data from March 2021 to December 2023. The main exposure variable was daily ST for each child, and tic severity, evaluated using the Yale Global Tic Severity Scale (YGTSS), was the outcome variable. Statistical analysis included descriptive statistics, Pearson’s correlation analysis to examine the relationship between screen time and tic severity, and multivariate regression analysis to evaluate the predictive power of screen time for tic symptoms.

**Results:**

Our findings revealed that children with TDs had significantly longer ST compared to the control group, averaging 116.06 ± 147.9 min/day versus 43.23 ± 37.5 min/day, *p* < 0.001. We also noted a positive correlation between ST and TDs( *r* = 0.461, *p* < 0.01). Daily ST was a significant predictor of overall YGTSS scores (t = 9.58, *p* < 0.001), suggesting that increased ST is associated with heightened tic symptoms. However, age of first exposure to screens was not significantly correlated with tic severity (*p* > 0.05). Though we observed a negative correlation between ST and vitamin D levels, the results were not statistically significant (*p* > 0.05).

**Conclusion:**

Children with TDs had longer ST compared to their control’s counterparts, and prolonged ST was significantly associated with heightened tic severity, which highlights the critical need for careful monitoring and regulation of screen time in children with TDs.

**Supplementary Information:**

The online version contains supplementary material available at 10.1186/s13052-025-01851-w.

## Background

Tic disorders (TDs) are a group of childhood neurodevelopmental conditions characterized by sudden, repetitive motor movements or vocalizations called “tics” [1]. They have been linked to congenital causes, including genomic mutations [2–4], as well as prenatal, perinatal, and neonatal risks [5–7]. Several factors exacerbate tic symptoms, including stress, exhaustion, stimulants like caffeine, and environmental factors such as heat [8, 9]. Prolonged screen time (ST) in childhood has emerged as a possible risk factor. While studies are yet to documented its effects on TDs, it has been shown to exacerbate symptoms of autism [10], attention-deficit/hyperactivity disorder (ADHD) [11] and other neurodevelopmental disorders [12]. As a result, prolonged ST, especially during childhood, a period of fast growth and development, could be one of the environmental factors that worsen tic severity.

Studies suggest that too much ST during childhood disrupts the formation of critical brain circuits necessary for inhibitory control [13]. This function, integral to the brain’s executive processes, is pivotal for regulating thoughts, emotions, and behaviors, enabling individuals to maintain focus and self-regulate despite strong temptations [14]. Children with extensive screen exposure commonly exhibit deficits in this aspect. Additionally, prolonged screen use correlates with a preference for immediate gratification over delayed outcomes, potentially interfering with the establishment of neural pathways crucial for inhibitory control [15].

In 2019, the World Health Organization issued guidelines on healthy physical activity, sedentary behavior, and sleep in children under the age of five, indicating that children at one year of age or younger should not be exposed to screens [16]. The American Academy of Pediatrics has also suggested that children not be exposed to screens until they are 18 months old, citing concerns about the negative effects of screen exposure on children’s health [17]. All of these are responses to the growing link between prolonged screen time and health problems in children.

Previous studies have shown that television and video games can exacerbate tic symptom [18–20]. Yet, the impact of ST on tic disorders remains unexplored. Additionally, prolonged ST has been linked to reduced outdoor activity [21], contributing to vitamin D deficiency [22], which may worsen TDs symptoms [23]. We thus undertook this study to examine the relationship between prolonged screen exposure (an environmental factor) and the severity of tic symptoms in children with TDs. We also investigated the relationship between ST and vitamin D levels in these children, considering that increased ST may reduce outdoor activity. We hypothesized that prolonged ST is associated with both worsened tic symptoms and reduced vitamin D levels. The findings of this study are expected to inform the development of effective management strategies for ST, thereby mitigating its harmful effects and enhancing the treatment and prevention of TDs in children.

## Methods

### Study design and participants

We conducted a retrospective case-control study at the Department of Developmental Behavioral Pediatrics of the First Hospital of Jilin University, Changchun, China. The study protocol was reviewed and approved by the First Hospital of Jilin University Research Ethics Committee. 342 children were recruited to participate in the study and informed consent was sought from parents or legal guardian of all of them. All the study participants met the DSM-5 (Diagnostic and Statistical Manual of Mental Disorders, Fifth Edition) diagnostic criteria for TDs. Retrieved medical records dated from March 2021 to December 2023. Of the 342 cases, 266 were boys and 76 were girls. Overall, they had a mean age of 7.76 ± 2.76 years. We also recruited 270 age-matched neurotypical children with a mean age of 7.43 ± 2.30 years to serve as a control group. These children underwent routine physical examinations in outpatient clinics and had comprehensive mental evaluations that confirmed they did not meet the DSM-5 diagnostic criteria for TDs or any other neurodevelopmental disorders. We excluded children who were diagnosed with other neurodevelopmental disorders, such as autism spectrum disorder (ASD), epilepsy, ADHD, or global developmental delay, and those with incomplete medical records.

### Collected data

The main exposure variable was mean daily screen time for each child, calculated as: mean daily screen time (minutes) = [(screen time per day on weekdays × 5) + (screen time per day on weekends × 2)] / 7. Daily ST was reported by parents through a self-filled questionnaire that captured a child’s ST duration in minutes per day on both weekdays and weekends. The responses were captured as continuous variables in minutes. All responses noted in hours were later converted to minutes [24]. The main outcome variable was tic severity among the children, evaluated using the Yale Global Tic Severity Scale (YGTSS), administered by experienced evaluators at the department who ensured rigorous quality control and standardized reporting [25, 26].

Recommendations by WHO explicitly prohibits screen exposure to children aged one year and below [21] in response to available evidence linking screen exposure in toddlers to neurodevelopmental challenges. Therefore, as a modifier variable, parents were to indicate the age (in months) at which the child was first exposed to a screen. We thus, collected information on vitamin D (25-hydroxyvitamin D [25(OH) D]) levels among the children. Concentrations of 30 to 90 ng/ml are considered optimal, between 10 ng/ml and 30 ng/ml are considered insufficient, while below 10 ng/ml is considered deficient [27]. Other important variables collected encompassed the children’s demographic information that included their age (in months), gender and daily outdoor activities. Data on ST also included type of electronic device used and primary screen activity engaged in.

We also gathered data on various subtypes of tic disorders as defined by the American Psychiatric Association. Tic disorders are categorized into three main subtypes: Transient Tic Disorder (TTD), Chronic Motor or Vocal Tic Disorder (CTD), and Tourette Syndrome (TS). TTD is characterized by the sudden onset of one or more motor or vocal tics, persisting for at least four weeks but not exceeding twelve months. CTD involves either motor or vocal tics (but not both) that last for more than a year. TS represents a more complex tic disorder, defined by multiple motor tics and at least one vocal tics [28].

### Statistical analyses

We used the Statistical Package for Social Sciences (SPSS), Version 27 (IBM) for all statistical analyses. First, we summarized the continuous variables of age, daily screen time, daily outdoor activity and YGTSS score inform of means and their corresponding standard deviations (SDs). Categorical variables were summarized inform of frequencies and percentages. Student’s t-tests was used to compare means of the case and control groups, while univariate ANOVA was used to compare means of more than two continuous variables. We then used the Pearson’s Chi-square test to compare categorical variables and the Pearson’s correlation coefficient (r) for correlation between ST and TDs. Lastly, we conducted a multivariate linear regression analysis, adjusting for age and gender, to determine the association between children’s ST and outdoor activities with YGTSS scores and vitamin D levels. Statistical significance was set as *p* < 0.05.

## Results

### Patient’s clinical characteristics

We observed similarity between the two groups in terms of age (*p* > 0.05). However, they significantly differed with regards to sex distribution, age of initial exposure to screens, daily ST, types of electronic screens exposed to, primary screen activity and daily outdoor activity (all *p* < 0.05). Children with TDs had significantly higher daily screen time than their control counterparts (116.06 ± 147.9 min/day vs. 43.23 ± 37.5 min/day; *p* < 0.000). They were also exposed to screens much earlier than controls and engaged in more screen-based activities. We also noted that children with TDs spent more time outdoors daily compared to the control group (80.90 ± 52.25 min vs. 65.50 ± 55.3 min). Details are presented in Table 1.

**Table 1:** Major characteristics of the participants

Variables	Cases (*n* = 342)	Controls (*n* = 270)	*P*-value
Age (years) M(SD)	7.76 (2.76)	7.43 (2.30)	0.116
Sex			
Male (%)	266 (77.8)	146 (54.1)	0.000
Female (%)	76 (22.2)	124(45.9)	
Daily outdoor activity M(SD)	80.90 (52.2)	65.50 (55.3)	0.000
Daily Screen time (min/day) M(SD)	116.07 (147.9)	43.23 (37.5)	0.000
Age of first screen exposure (months)	25.92 (11.4)	28.86 (12.0)	0.002
Type of electronic Screens			
Television, mobile phones and tablets (%)	340 (99.4)	222 (82.2)	0.000
Others (%)	2 (0.6%)	48 (17.8)	
Main activities of the electronic screen			
Entertainment (%)	297 (86.8)	201 (74.4)	0.000
Educational (%)	38 (11.1)	55 (20.4)	
Others (%)	7 (2.0)	14 (5.2)	
YGTTS score			
Total YGTSS scores M(SD)	31.30 (10.11)	---	---
Total tics scores M(SD)	19.13 (7.41)	---	---
Motor tics M(SD)	12.48 (5.13)	---	---
Phonic tics M(SD)	6.86 (5.90)	---	---
Impairment M(SD)	12.08 (4.86)	---	---

### Association between prolonged ST and Tic severity

Pearson’s correlation coefficients revealed a significant positive correlation between mean daily ST and total YGTSS score (*r* = 0.461, *p* < 0.01), indicating that mean daily ST was associated with symptoms of tic disorder Table 2. Similarly, a significant positive correlation was observed between ST and the severity of symptoms across different TD subtypes, with the strongest correlation noted in CTD (*r* = 0.778, *p* < 0.01) Supplementary material 1. Furthermore, multivariate linear regression analysis showed that mean daily ST positively predicted total YGTSS score (t = 9.58, *p* < 0.001), suggesting that increased daily ST correlated with heightened tic severity Table 3.

**Table 2:** Correlation between screen time and tic disorders (results of Pearson correlation)

Pearson’s correlation	*R*	*P*
YGTSS score	0.461	< 0.001
Motor score	0.248	< 0.001
Vocal score	0.250	< 0.001
Total tic score	0.381	< 0.001
Impairment	0.369	< 0.001

**Table 3:** Multivariate linear regression analysis predicting YGTSS scores based on daily screen time and age of first screen exposure

Coefficients^a^						
	B(SE)	β	t	P	R^2^	95% CI
Daily screen time	0.032 (0.003)	0.463	9.58	0.000	0.213	0.025– 0.038
Age of first screen exposure	0.021(0.043)	0.024	0.494	0.621		-0.063–0.105

a. Dependent Variable: YGTSS score

### Association between age of first screen exposure and tic severity

In the multivariate linear regression analysis, we also assessed the age of first exposure to screen and severity of tic symptoms. The results indicated no significant relationship between the age of first screen exposure and the YGTTS score (t = 0.494, *p* > 0.05) Table 3. Additionally, no significant correlations were found between the age of first screen exposure and symptom severity across different tic disorder subtypes (all *p* > 0.05) Supplementary material 2.

### Serum vitamin D levels, daily ST and outdoor activity in children with tic disorders

**Fig. 1:**
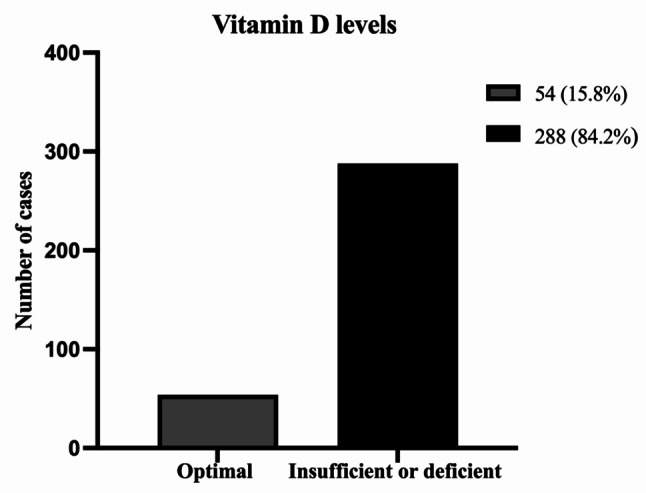
Serum vitamin D levels in children with tic disorders

Regarding serum vitamin D levels in children with TDs, our findings indicate that the majority of children displayed insufficient or deficient levels of vitamin D, with only 15.8% achieving optimal levels, as illustrated in Fig. 1. Further analysis of vitamin D levels across TD subtypes—including TTD, CTD, and TS—revealed no statistically significant differences (all *p* > 0.05). Specifically, 84.4% of TTD cases, 75.5% of CTD cases, and 88.3% of TS cases exhibited insufficient or deficient vitamin D levels. We also evaluated daily ST and outdoor activity among the three subtypes, finding no significant differences (all *p* > 0.05) Supplementary material 3.

### Associations among daily ST, outdoor activity, vitamin D level and tic severity

Lastly, we conducted multivariate linear regression analyses to predict vitamin D levels based on daily ST and daily outdoor activities, adjusting for age and gender. The results were both not statistically significant (all *p* > 0.05), although a positive association was noted between daily outdoor activities and vitamin D levels (t = 0.879), and negative association between daily ST and vitamin D levels (t = -1.256) Table 4. In the subsequent regression analysis, we assessed the association between outdoor activities, vitamin D levels, and tic severity. Again, a negative association was observed between outdoor activities, vitamin D levels and tic severity; however, these associations were not statistically significant (t = -0.749 and t = -0.871, *p* > 0.05) Table 5.

**Table 4:** Multivariate linear regression analysis to predict vitamin D levels based on daily outdoor activities and daily screen time

Coefficients^a^						
	B(SE)	β	t	P	R^2^	95% CI
Daily outdoor activities	0.011(0.013)	0.048	0.879	0.380	0.007	-0.014–0.037
Daily screen time	-0.006(0.005)	-0.068	-1.256	0.210		-0.015–0.003

a. Dependent Variable: Vitamin D levels

**Table 5:** Multivariate linear regression analysis predicting YGTSS scores based on daily outdoor activities and vitamin D levels

Coefficients^a^						
	B(SE)	β	t	P	R^2^	95% CI
Daily outdoor activities	-0.008 (0.011)	-0.041	-0.749	0.454	0.004	-0.029–0.013
Vitamin D levels	-0.038 (0.044)	-0.047	-0.871	0.384		-0.124–0.048

a. Dependent Variable: YGTSS score

## Discussion

The main finding of this study was that prolonged ST was significantly associated with severity of tic symptoms. Multivariate linear regression analysis showed that increase in daily ST among children with TD resulted in increased YGTSS scores, indicating worsened tic symptoms. This finding is supported by previous studies that found connection between watching television and video games to worsened tic symptoms [18–20]. Similar connections have been observed in related neurodevelopmental disorders such as ASD and ADHD [10, 29–32]. A plausible explanation for the observed relationship lies in the neurobiological mechanisms being triggered by ST. Excessive ST may worsen inhibitory control deficits that exist in TDs, thus hindering the suppression of unwanted movements or behaviors [33, 34].

Several studies have linked excess dopamine release to tic like behaviors [35–37]. Screen based activities, characterized by repetitive actions such as tapping or scrolling are models for tic-like behaviors in susceptible individuals [38]. Fast-paced video games and captivating animations are engineered to engage users by triggering dopamine release and influencing the brain’s reward system [39]. This reduces inhibitory control in children resulting into worsening tic symptoms. Similarly, prolonged exposure to screen-based stimuli, featuring fast-paced visual and auditory input are likely to overwhelm the brain’s sensory processing mechanisms, leading to increased arousal levels and stress responses [40], which are known to worsen tic symptoms and may amplify existing vulnerabilities in children with TDs [35].

In addition to genetic factors, the role of environmental factors in TDs are prominent. Electromagnetic fields have been cited as an environmental factor associated with health and screen exposure [41]. Experiments using mice have demonstrated that exposure to high-frequency electromagnetic fields affects neurotransmitters [42] and behavior (hyperactivity and memory impairment) during the developmental period [43]. Additionally, tic disorder is associated with a significant loss of inhibitory control in the brain. Tic behaviors are caused by the failure of the cortico-striato-thalamocortical circuits to suppress somatosensory urges and accompanying motor activity [44–47]. Importantly, tics are considered a focal excitatory anomaly in the striatum, generating greater inhibition of the globus pallidus internus, resulting in disinhibition of the thalamic and, in turn, cortical neurons [48, 49]. For instance the “premonitory urge,” that precedes a tic reflects a deficient inhibitory control over the motor response to premonitory urge, which evidence suggest is due to disruptions in movement-regulation functions mediated by the basal ganglia [50]. This disruption has been shown under both laboratory and natural conditions to be influenced by environmental and behavioral factors such as stress, being in public, anxiety, and screen-based activities (e.g., watching television), among others [18, 19, 51].

Previous neurodevelopmental research has shown that the influence of ST on a child’s brain development is most noticeable very early on [52, 53]. Toddlers under one year old are the most exposed to the harmful effects of electromagnetic waves. In this study, children with TDs were exposed to screens at a much younger age than the controls, with a mean age of 25.92 ± 9.75 months versus 28.86 ± 12.0 (*p* < 0.05). However, we found no significant association between the age of first screen exposure and the severity of tic symptoms. Furthermore, vitamin D administration has been demonstrated to alleviate the severity of tic symptoms [54]. However, in this study, we found no association between vitamin D levels and tic severity. This finding is consistent with results by Wang et al., [55] among Chinese children and Bond et al., [56]. among European children. We also found no difference in the levels of vitamin D among the different subtypes of TDs. This is consistent with the findings of a meta-analysis by Xiaoxia et al., [57]. Future well-designed randomized controlled trials are needed to study the therapeutic potential of physical activity and vitamin D supplementation in the treatment of tic disorders.

To the best of our knowledge, this study represents the first attempt to explore the correlation between daily screen time and TDs. While providing significant insights into their potential relationship, our study has certain limitations worth mentioning. Firstly, it shares the common drawbacks of retrospective studies, such as dependence on self-reported screen time, which may lead to recall bias and measurement inaccuracies. Secondly, the absence of vitamin D data among control subjects, combined with the known association between TDs and low levels of vitamin D, complicates our ability to definitively establish a direct link between low vitamin D levels and tic disorders. Future studies that take a prospective approach to address these limitations will be crucial for enhancing our comprehension of the interplay between screen time, vitamin D levels, and tic disorders. Additionally, clinicians should design individualized screen time plans, integrating these strategies into behavioral therapy and educating parents on effective management techniques. Public health initiatives, including awareness campaigns and school policies, are crucial for promoting balanced screen use.

## Conclusions

In summary, this research discovered children with TDs spent more time on screens than their neurotypical counterparts, and prolonged ST was associated with heightened tic severity. However, there was no significant relationship between age of first screen exposure and severity of TDs. While we observed a negative correlation between ST and vitamin D levels, the finding was not statistically significant. These findings will guide the creation of successful approaches to managing ST in children with TDs, thus reducing its adverse impacts on tic severity and improving the prevention and treatment of TDs.

## Electronic supplementary material

Below is the link to the electronic supplementary material.

Supplementary Material 1

Supplementary Material 2

Supplementary Material 3

## Data Availability

The datasets used and analyzed during the current study are available from the corresponding author on reasonable request.

## Abbreviations

ST: Screen time
TDs: Tic disorders
TTD: Transient Tic Disorder
CTD: Chronic Motor or Vocal Tic Disorder
TS: Tourette Syndrome
ASD: Autism spectrum disorder
ADHD: Attention-deficit/hyperactivity disorder
YGTSS: Yale Global Tic Severity Scale
DSM-5: Diagnostic and Statistical Manual of Mental Disorders, Fifth Edition
