# Supplementary material for: Prolonged screen time is associated with increased severity of tic symptoms in children with tic disorders
Source: Ital J Pediatr. 2025 Jan 26;51:16. doi: 10.1186/s13052-025-01851-w (PMC11770938; doi:10.1186/s13052-025-01851-w)
Supplement: Supplementary file 1 — Supplementary Material 1 [file 13052_2025_1851_MOESM1_ESM.docx]

**Table 1.** Association between screen time and TD subtypes (results of Pearson correlation)

| Pearson’s correlation | R | P |
| --- | --- | --- |
| TTD | 0.396 | <0.001 |
| CTD | 0.778 | <0.001 |
| TS | 0.463 | <0.001 |

TTD; Transient tic disorder, CTD; Chronic tic disorder, TS; Tourette syndrome
