# Supplementary material for: Prolonged screen time is associated with increased severity of tic symptoms in children with tic disorders
Source: Ital J Pediatr. 2025 Jan 26;51:16. doi: 10.1186/s13052-025-01851-w (PMC11770938; doi:10.1186/s13052-025-01851-w)
Supplement: Supplementary file 2 — Supplementary Material 2 [file 13052_2025_1851_MOESM2_ESM.docx]

| Coefficients^a^ | | | | | | |
| --- | --- | --- | --- | --- | --- | --- |
|  | **B(SE)** | **β** | **t** | **P** | **R^2^** | **95% CI** |
| TTD | 0.033(0.066) | 0.037 | 0.505 | 0.614 | 0.001 | -0.097-0.164 |
| CTD | -0.181(.099) | -0 .248 | -1.827 | 0.074 | 0.061 | -0.380-0.018 |
| TS | -.021(0.080) | -0.027 | -.268 | 0.789 | 0.001 | -0.180-0.137 |
| a. Dependent Variable: YGTSS score | | | | | | |

**Table 2.** Multivariate linear regression analysis predicting YGTSS scores based on age of first screen exposure by TD subtypes

TTD; Transient tic disorder, CTD; Chronic tic disorder, TS; Tourette syndrome
