# Supplementary material for: Prolonged screen time is associated with increased severity of tic symptoms in children with tic disorders
Source: Ital J Pediatr. 2025 Jan 26;51:16. doi: 10.1186/s13052-025-01851-w (PMC11770938; doi:10.1186/s13052-025-01851-w)
Supplement: Supplementary file 3 — Supplementary Material 3 [file 13052_2025_1851_MOESM3_ESM.docx]

**Table 3.** Serum vitamin D levels, daily screen time, and daily outdoor activities in children with different subtypes of tic disorders

| Variables | TTD (186) | CTD (53) | TS (103) | Total (342) | F | *P-*value |
| --- | --- | --- | --- | --- | --- | --- |
| Vitamin D status | | | | | | |
| Optimal (%) | 29 (15.3) | 13 (24.5) | 12 (11.7) | 54 (15.8) |  | 0.12 |
| Insufficient or deficient (%) | 157 (84.4) | 40 (75.5) | 91 (88.3) | 288 (84.2) |  |  |
| 25(OH)D, (ng/ ml) | 21.65 ± 12.09 | 24.04 ± 13.09 | 21.68 ± 12.97 |  | 0.808 | 0.446 |
| Daily screen time | 104.07 ± 134.14 | 124.85 ± 150.10 | 133.19 ± 168.67 |  | 1.397 | 0.249 |
| Daily outdoor activities | 81.43 ± 52.32 | 88.86 ± 57.11 | 75.83 ± 49.41 |  |  |  |

TTD; Transient tic disorder, CTD; Chronic tic disorder, TS; Tourette syndrome
